# Evaluating main gas emission and energy consumption economy during tobacco leaf curing life cycle based on clean energy

**DOI:** 10.1038/s41598-025-98956-3

**Published:** 2025-04-22

**Authors:** Yinlong Zhu, Yonglei Jiang, Yi Chen, Jiaen Su, Ming Li, Qiongfen Yu, Zhenhua Gu, Jianhuan Deng, Sheng Tang, Zhihao Song, Xudong Wang, Bo Ma

**Affiliations:** 1 Yunnan Academy of Tobacco Agriculture Science, Kunming, 650300 Yunnan China; 2 School of Energy and Environment Science, Yunnan Normal University, Kunming, 650500 Yunnan China; 3 Key Laboratory of Solar Heating and Cooling Technology of Yunnan Provincial Universities, Kunming, 650500 Yunnan China; 4 Southwest United Graduate School, Kunming, 650500 Yunnan China

**Keywords:** Clean Energy, Tobacco curing, Life cycle, Main gas emissions, Energy economy assessment, Climate sciences, Ecology, Environmental sciences, Environmental social sciences, Energy science and technology

## Abstract

Coal roasters suffer from unstable combustion, resulting in high energy consumption and environmental pollution, this paper explores main gas emissions and energy consumption economy of biomass, alcohol-based and natural gas ovens in order to obtain the best alternative energy source. The paper conducted a comprehensive analysis of the following aspects in clean energy tobacco curing: major gas emission concentrations and volumes at each stage, emission quantities per cubic meter of major gases, gas emissions during the leaf yellowing/color-fixing stage and stem-drying stage of tobacco curing, total lifecycle emissions of major gases and energy consumption efficiency/economic feasibility of the curing facility. Data analysis showed that CO_2_ emission concentration of each energy roaster reached the highest in all stages under standard working conditions, and CO_2_ emission of biomass roaster reached 550.68 kg in the 10th stage, while there was no SO_2_ emission from alcohol-based and natural gas roasters. In addition, the highest total emissions per cubic meter of each of the major gaseous emitters CO_2_, SO_2_, NO and NO_2_ were all biomass, 21.1 kg, 0.057 kg, 0.036 kg and 0.0005 kg, respectively. During the baking process, CO_2_ emission of natural gas per ton of wet tobacco leaves was only 105.97 kg, with no NO and NO_2_ emissions, and no SO_2_ emissions from alcohol-based and natural gas. The emissions of CO_2_, SO_2_, NO and NO_2_ increased gradually in the yellowing period, color fixation period and dry tendon period, and most emissions were in the dry tendon period of biomass roasting room, which were 1203.85 kg, 35.23 kg, 1.53 kg and 0.248 kg, respectively. There was no emission of SO_2_ in the alcohol-based and natural gas roasting rooms. In addition, biomass had the largest total emissions in the entire life cycle of clean energy tobacco leaf baking. As baking time increases, pollutant emissions also increase. The highest CO_2_ emissions from biomass were 1518.82 kg. CO_2_ emission rates of alcohol-based and natural gas baking rooms are relatively slow, and the minimum CO_2_ emissions from the natural gas baking room were 582.82 kg. The ratio of fresh and dry cigarettes after roasting was roughly around 7:1, with the least consumption of natural gas for fresh tobacco, lower power consumption and a higher average dehumidification ratio per unit of energy consumption. The unit cost of dried tobacco leaves in the biomass curing room was 1.35 yuan, which was 22.3% and 16.2% lower than alcohol-based and natural gas. Although biomass curing barns had the lowest unit cost for dried tobacco leaves, their combustion produces more gas emissions. These results showed that biomass had the largest number of major gas emissions and the largest emission volumes, while natural gas and alcohol genes had lower emissions and better economic performance, which can play a significant role in energy conservation, emission reduction, cost reduction and efficiency improvement.

## Introduction

Currently, the world is facing the double challenge of energy crisis^1,2^ and environmental pollution^3,4^. Coal consumption, as the traditional energy source for tobacco roasting^5^, amounts to about 3–4 million t per year, with carbon dioxide emissions close to 8 million t, soot of about 600,000 t, and 30–50 million t of harmful gases, producing a large amounts of solid dust particles^6^. This resulted in low heat utilization^7^, unstable combustion, and serious environmental pollution^8^. New energy roasting room is undoubtedly a future trend for tobacco roasting in China. In October 2021, the State Tobacco Monopoly Administration issued the "14th Five-Year Plan" green, low-carbon and circular development work plan policy, which clearly proposed to promote mature clean energy baking technology, aiming for green transformation of tobacco production and the “dual carbon” goal. In November 2024, the “new energy” clean baking in the Luzhou tobacco area of ​Sichuan Province demonstrated its potential for producing high-quality tobacco leaves. In September 2023, similar attempts have been made in Kunming Tobacco Monopoly Bureau (Company), promoting energy-saving tobacco leaf baking. These attempts policies facilitated the gradual transition from biomass to clean fuels in tobacco baking process. At present, new energy sources used for tobacco roasting mainly include biomass fuel, heat pump, solar energy, methanol fuel, natural gas and so on^9,10,11^. Introduction of these resources, along with a comprehensive assessment of major gas emissions in tobacco roasting in different stages, gradually moved the industry toward a more efficient, sustainable and environmentally friendly production mode^12^.

In recent years, domestic and foreign scholars have proposed a variety of methods for reducing carbon emissions and energy consumption in tobacco leaf baking. First, energy substitution is a common carbon reduction measure^13^. Natural gas can significantly reduce carbon dioxide emissions, but the acquisition and transportation costs are high^14^. Alcohol-based fuel has the advantage of a accessible raw materials, low cost and no pollution^15^. Biomass resources are abundant and carbon emissions are relatively low, but a certain amount of pollutants will still be produced during the combustion process^16^. In view of the characteristics of the above-mentioned clean energy sources, Zhang et al.^17^ studied the application technology of biomass to replace coal and found that it can effectively reduce baking costs, improve baking efficiency, and reduce pollution. It has great potential to replace coal as the major baking fuel. Du et al.^18^ found that biomass pellets replacing coal in tobacco leaf baking has obvious cost reduction, efficiency improvement and environmental protection effects. Huang et al.^19^ found that compared to coal-fired curing rooms, alcohol-based curing rooms embody obvious advantages in reducing pollution and carbon emissions, improving the quality and efficiency of tobacco leaf baking. Jia et al.^20^ studied the thermal characteristics of natural gas tobacco baking systems and found that natural gas baking rooms have a high degree of automation, significantly reduced labor costs, and the total cost of 1 kg of dry tobacco leaves is 0.32 yuan lower than that of coal-fired baking rooms, demonstrating significant economic benefits and energy conservation effects. In sum, the above research and analysis found that each clean energy has its own advantages in replacing coal as a tobacco baking fuel. Secondly, use of intelligent control systems for real-time monitoring and adjustment can accurately manage the baking process, thereby reducing energy consumption and emissions^21^. In addition, the operating cost of heat recovery technology in tobacco baking is also relatively low, but it requires modifying existing equipment, and the initial investment is relatively large^22^.

The above tobacco leaf curing methods all have their own advantages, but biomass, alcohol-based, and natural gas are currently the most suitable curing methods rather than coal^23^. Comparison of main gas emissions and energy economy of tobacco leaf under different energy methods, enables the selection of more economical, environmentally friendly, and efficient energy to achieve sustainable development. Lan Shubin et al.^24^ found that for every ton of crop straw burned, 791.3 kg of CO_2_, 0.53 kg of SO_2_, and 1.29 kg of NO_X_ will be emitted, and the cost is 1.29 yuan, while for every ton of coal burned, 2,620 kg of CO_2_, 8.5 kg of SO_2_, and 7 kg of NO_X_ will be emitted, and the cost is 1.47 yuan. This shows that using biomass curing rooms can reduce fuel consumption and tobacco curing costs. Zheng Dongfang et al.^25^ compared the differences in energy consumption, labor, environmental protection and economic benefits between biomass curing houses and traditional coal-fired curing houses in Chuxiong Prefecture, Yunnan Province, and found that comprehensive energy consumption of biomass curing houses increased by 0.40 yuan/kg. The labor cost of flue-cured tobacco decreased by 0.27 yuan/kg and emissions of CO_2_, SO_2_ and CO were all reduced. Yang Zhiren et al.^26^ explored the effects of coal, biomass, natural gas and electricity on CO_2_ emissions during flue-cured tobacco production in Chongqing, and found that replacing coal with biomass energy can reduce CO_2_ emissions by 32%. Replacing coal with natural gas can reduce carbon emissions by 52%, and replacing coal with electricity can achieve up to 69% of carbon emission reduction. Tan Fangli et al.^27^ compared and analyzed differences in energy consumption, temperature stability performance and economic benefits between biomass, methanol, air source heat pump and coal-fired curing houses, and found that alternative energy curing houses are superior to coal-fired curing houses in improving tobacco leaf quality and temperature control. Guo Dayang et al.^28^ compared and analyzed main gas emissions of biomass molded fuel, biomass pellets, alcohol-based and conventional lignite in tobacco leaf baking. From CO_2_ emissions, it can be seen that the CO_2_ content in flue gas of alternative energy combustion is 0.3–0.4 percentage points higher than that of the lignite. From the emission of other major pollutants, it can be seen that CO and SO_2_ emissions of alternative energy combustion are significantly lower than those of lignite, and the emission reduction effect is obvious. Guo Weimin et al.^29^ collected baking temperature and humidity data of three tobacco areas in Henan Province through IoT data. The yellowing period, color fixation period and dry tendon period in the baking process were analyzed and found that the yellowing period, color fixation period and dry tendon period accounted for about 40%, 35% and 25% of the total baking time in western and southern Henan, and the yellowing period accounted for about 55% of the total baking time in central Henan. Zhang et al.^30^ used IoT technology to collect temperature and humidity data of the baking room during the baking process from 2019 to 2021, and found that the baking process lasted longer regarding total time and yellowing period, and shorter in terms of color fixation and dry tendon period. Liu et al.^31^ conducted a comparative test on tobacco leaf baking technology in air-downward baking barns based on the actual situation of ecological baking barns in Jiangxi Province with high rainfall. They found that the use of multi-point heating and temperature stabilization during the yellowing and color fixation stages would cause the tobacco leaves to turn yellow and dehydrate during baking, leading to the shrinkage of rolls, and thus reducing the quality of tobacco leaves.

**Fig. 1:**
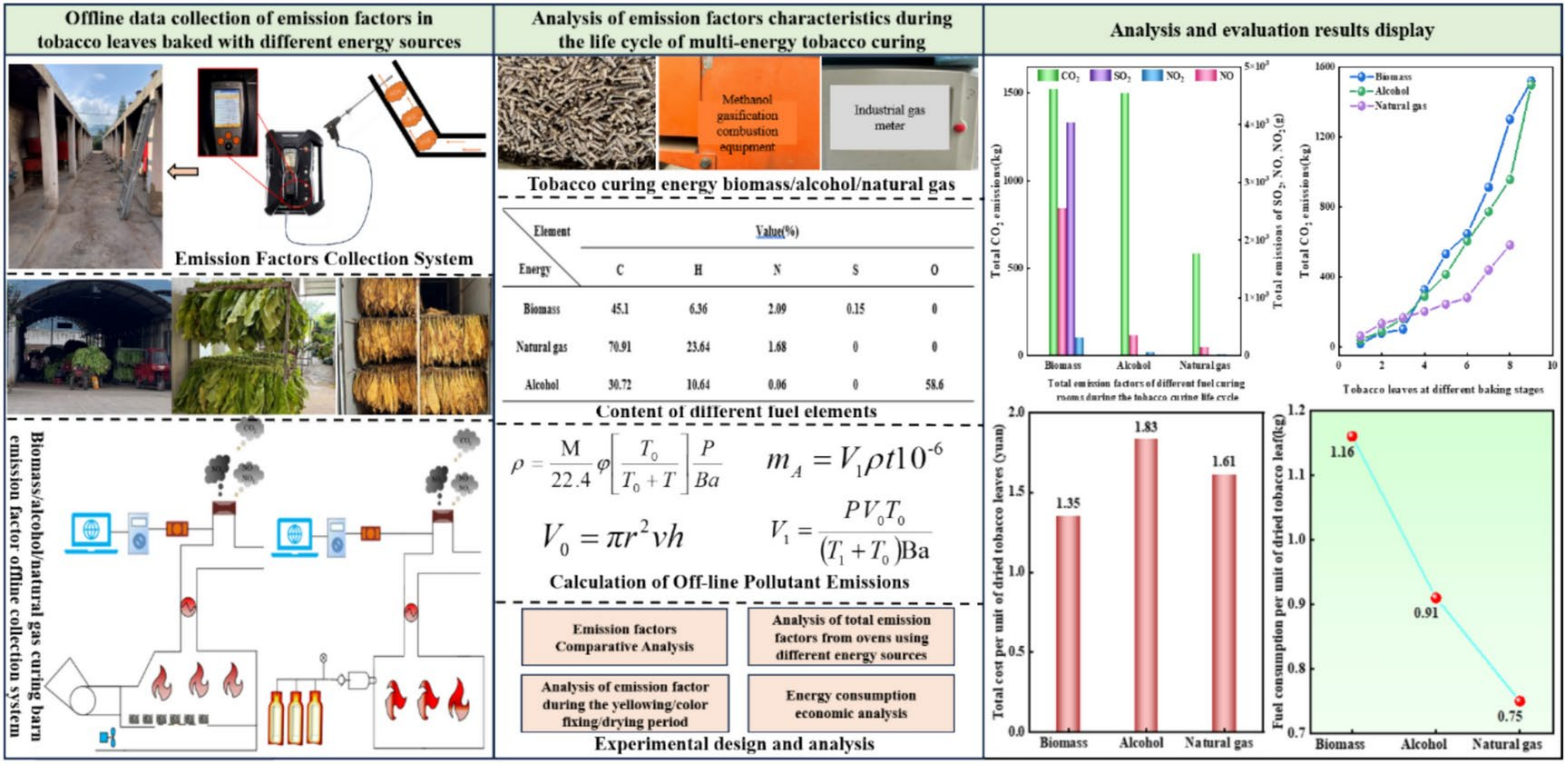
The framework for research on the evaluation of main gas emissions during tobacco leaf curing life cycle and energy consumption economy based on clean energy.

Overall, clean energy use in the tobacco leaf curing process has great advantages in terms of short-term investment, rapid results, energy conservation and environmental protection. Yet, as far as we know, in the field of tobacco leaf curing, few research calculated main gas emission concentration and amount at each stage, unit emission amount/cubic meter, and unit dehumidification energy consumption ratio^12,24,25^. There also lacks a comprehensive analysis of main gas emissions and energy economy during the yellowing period, color fixing period, and drying period of the curing process using various energy sources^26,28,30^. Therefore, this study uses statistical modeling to compare and analyze main gas emissions and emissions at each stage of the curing life cycle of biomass, alcohol-based and natural gas fuel curing rooms. At the same time, main gas emissions during the yellowing, color fixing and drying stages of tobacco leaf curing drawing from each energy source are statistically analyzed, and their energy consumption and economy are evaluated. One major highlight of this study is that for the first time, main gas emission concentration and emission amount, unit cubic meter emission, and unit dehumidification energy consumption ratio at each stage of the clean energy flue-cured tobacco life cycle are statistically analyzed. It is also pioneering in conducting a comprehensive analyses of main gas emissions during the yellowing, color fixing and drying stages of tobacco leaf baking under clean energy, which can enhance our understanding of the pollutant emission levels and sources at each stage and promote more sustainable and energy-conserving tobacco production. The research framework for evaluating main gas emissions and energy consumption in the tobacco leaf baking life cycle based on clean energy is shown in Fig. 1.

## Materials and methods

### Materials

**Fig. 2:**
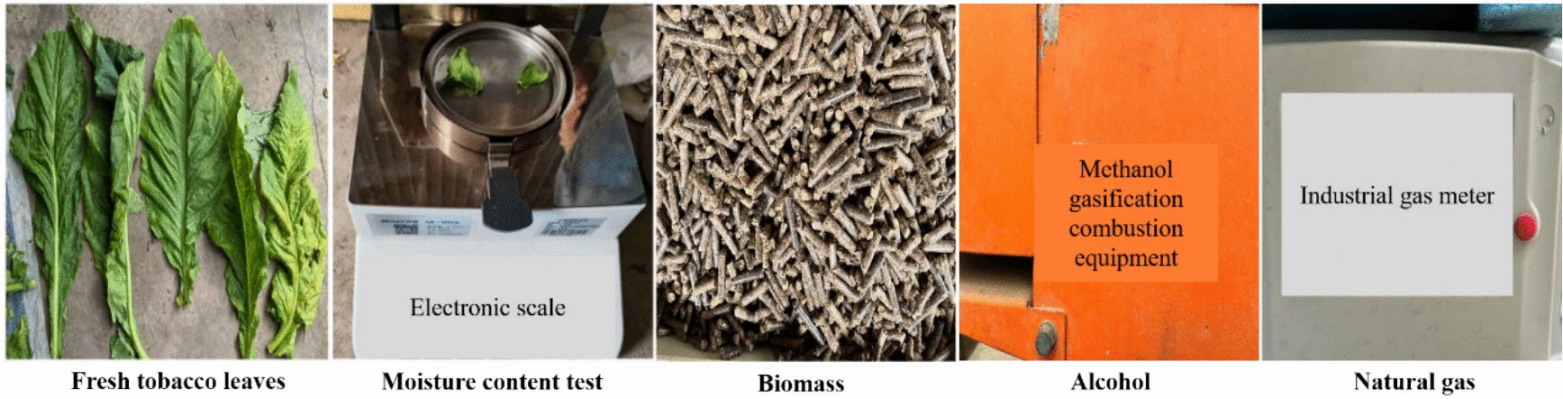
Schematic diagram of three energy sources and moisture content of fresh tobacco leaves.

This test site has more than 100 industrialized roasting houses, providing all types of fuel and supporting equipment required for the test. All the roasting rooms were 8 m × 2.7 m × 3.5 m, with the use of tobacco pole braiding, and the type of roasted tobacco was safflower Dajinyuan, with an average moisture content of 87.08%. This study takes drying one furnace of fresh tobacco leaves as a complete baking cycle, using biomass, natural gas and alcohol as energy sources. Among them, biomass fuel is compressed from straw, natural gas fuel uses bottled liquefied petroleum gas and alcohol-based fuel uses liquid methanol. The schematic diagram of the three energy sources used and the moisture content of fresh tobacco leaves is shown in Fig. 2.

By analyzing effective components of solid fuels using an elemental analyzer, it can be found that there are obvious differences in the elemental composition of different fuels. Natural gas has the highest C and H content, biomass has the highest N and S content, natural gas and alcohols do not contain S elements, and biomass and natural gas do not contain O elements. However, S and N elements are used to evaluate the environmental impact of energy, so more pollutants will be emitted during biomass combustion. In contrast, the N and S content of alcohols and natural gas remains within environmentally friendly levels. The element content of different fuels is shown in Table 1.

**Table 1:** Element content of different fuels.

Element Energy	Value(%)				
	**C**	**H**	**N**	**S**	**O**
Biomass	45.1	6.36	2.09	0.15	0
Natural gas	70.91	23.64	1.68	0	0
Alcohol	30.72	10.64	0.06	0	58.6

### Combustion system introduction

**Fig. 3:**
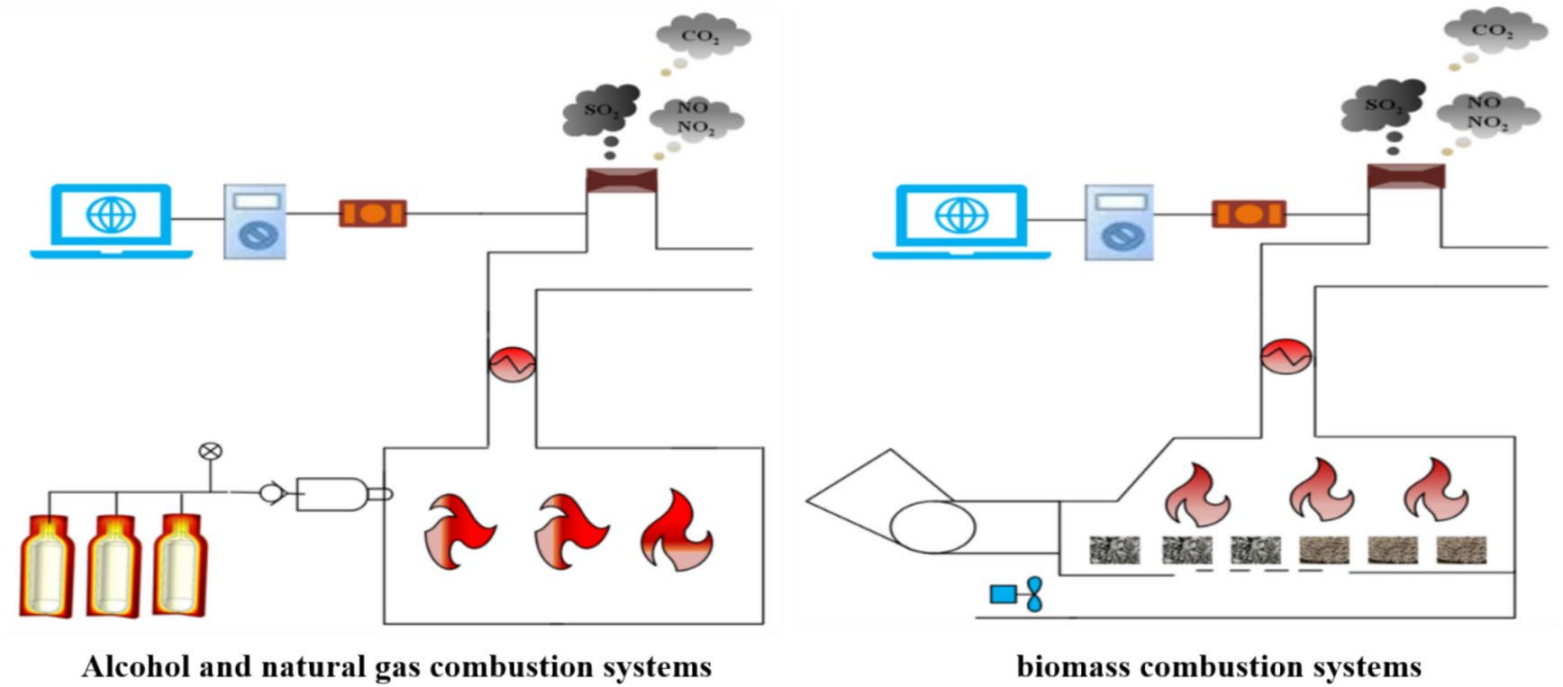
System working principles and physical diagrams of three different combustion modes.

The biomass system is mainly composed of a feeder, an induced draft fan, a burner and a heat exchanger. First, the fuel is automatically fed into the combustion chamber through the feeder for combustion. Then, the induced draft fan feeds fresh air into the combustion chamber to promote complete combustion of the fuel. Finally, the air in the curing room is heated by the heat exchanger, and the exhaust gas is discharged through the exhaust pipe. The gas fuel (natural gas, alcohol) system feeds the gas into the combustion tank through a pipeline and ignites it through an electric spark. Finally, hot air heats the air in the curing room through the heat exchanger, and exhaust gas is discharged through the exhaust pipe. The exhaust pipe is equipped with temperature, pressure and flow monitors to accurately monitor the flue gas flow in each curing room. In addition, for the measurement of CO_2,_ SO_2_ and NO_X_ concentrations in the flue gas emitted from the curing room, the collected flue gas is first sampled by the Testo 350i pollutant monitoring system. Then, the sampled flue gas is filtered out of impurities such as smoke oil carried in the flue gas by the pretreatment device, and then subjected to secondary treatment by the drying tube. Finally, it is fed into the Testo 350i through the air intake pipe. The system working principle and physical diagram of the three different combustion modes are shown in Fig. 3.

### Introduction to gas emissions collection system

Natural gas, alcohol-based and biomass fuel curing rooms were designed as experimental curing rooms. The concentrations of CO_2_, NO, NO_2_ and SO_2_ in the flue gas as well as the smoke temperature, smoke flow rate and actual pressure of the three fuel curing rooms were measured at each curing stage during their whole life cycle. Among them, the biomass and alcohol-based curing rooms have ten stages, the natural gas curing room has nine stages and the measurement method is once every hour. During the whole baking cycle, the number of measurements (sample size) of the biomass baking room was 213 times The number of measurements (sample size) of the alcohol-based baking room was 205 times, and the number of measurements (sample size) of the natural gas baking room was 258 times. The combustion time and measurement time of each stage of the three fuel baking rooms are shown in Table 2.

**Table 2:** The burning time and measurement time of each stage of the three fuel baking rooms.

Stage	Burning time			Measuring time
	Biomass	Alcohol	Natural gas	
1	5	5	5	Measure every 1 h
2	14	15	35	
3	34	40	82	
4	16	18	29	
5	21	21	24	
6	18	17	26	
7	24	22	19	
8	21	21	17	
9	20	18	21	
10	40	28		

**Fig. 4:**
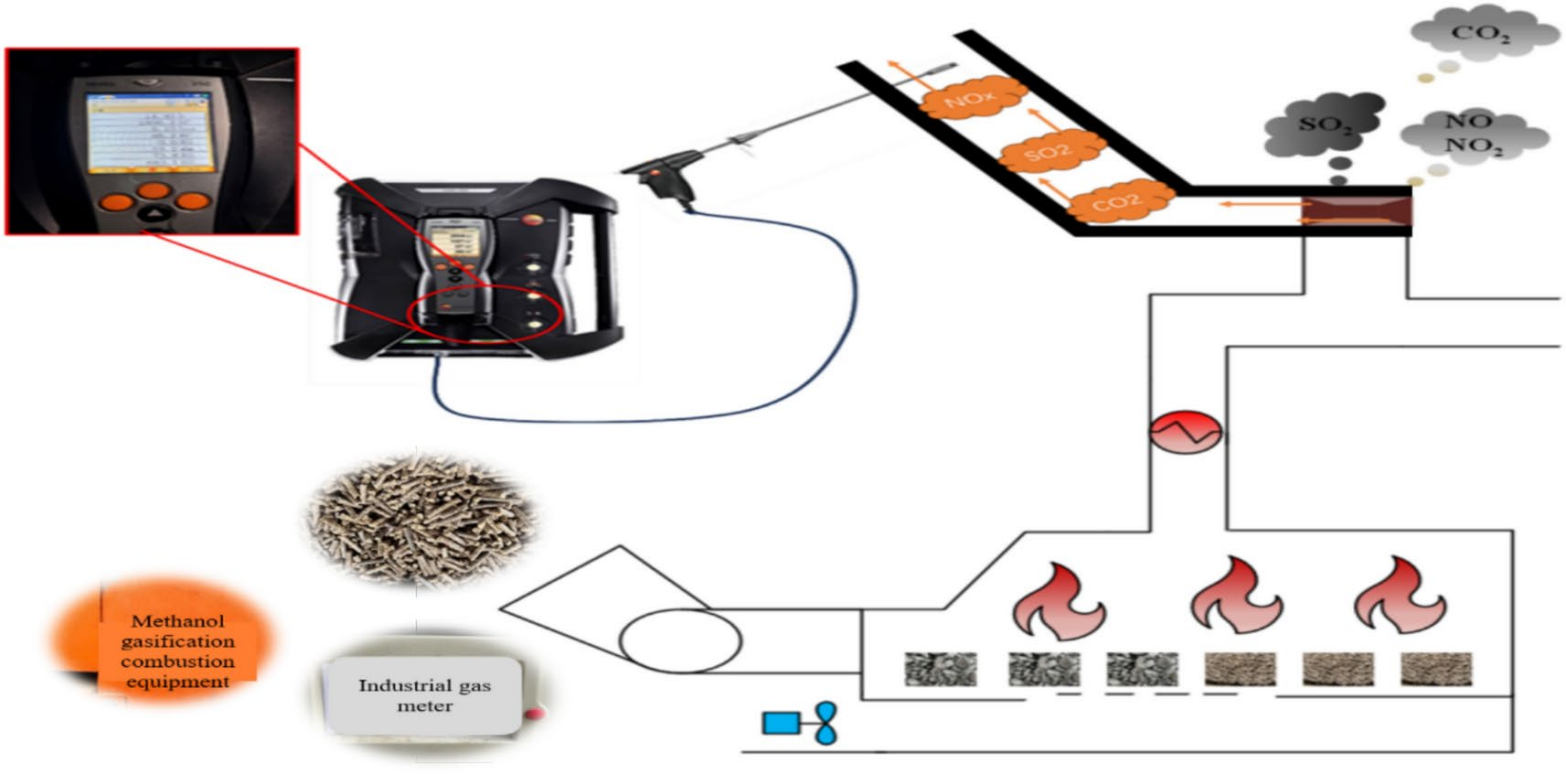
Testo 350i flue gas analyzer operating principle diagram.

The emission factor test system uses the Testo 350i portable flue gas analyzer to measure the gas components in flue gas through electrochemical sensors. Before measuring, we need to calibrate the instrument. First, check the instrument status to ensure that the power supply is normal, the sensor and gas system are not blocked, and the dust filter, condensate tank and other components are kept clean. Then preheat the instrument to a stable working state (15 min), prepare the standard gas using high-purity zero gas (nitrogen) and range gas (clean air with O_2_), and ensure that the standard gas concentration covers the measurement range of the instrument. Finally, perform zero calibration. Switch the instrument to the gas measurement mode to be calibrated (such as O_2_ mode), introduce zero gas, wait for the reading to stabilize, press the calibration key (SET key), select the “zero calibration” mode (ZERO light is on), confirm that the reading returns to zero, and save the calibration result. Then perform span calibration, introduce standard span gas (clean air with O_2_), ensure the flow is stable, wait for the instrument reading to stabilize, select the “Span Calibration” mode (SPAN light is on), enter the actual concentration value of the standard gas, and save the calibration result after confirmation. Repeat the zero point and span calibration three times. If the error does not exceed ± 5%, the instrument calibration is completed. During the experiment, holes were drilled in the middle of the exhaust ducts of three baking rooms with different fuels, and the concentrations of CO_2_, NO, NO_2_, SO_2_, as well as the smoke temperature, moisture content, smoke flow rate and actual pressure in the flue gas at each baking stage were measured using the probes of flue gas analyzer and the multi-functional humidity detector. Then, the flow rate is calculated by measuring the flue diameter and combining it with flow velocity. Then the total amount of various emissions is calculated, and the total amount of emissions is converted into mass under standard conditions (0℃, 101.325 kPa). The working principle of the Testo 350i flue gas analyzer is shown in Fig. 4.

### Introduction to the calculation method of gas emission

For the pollutant data collected by the above offline system, the pollutant emissions are counted through gas concentration unit conversion, hourly flue gas flow calculation, flue gas flow conversion to flue gas flow under standard conditions and total emission calculation of the measured pollutant concentration within a certain period of time, thus completing the unified processing of the calculation of pollutant emissions from drying rooms with different energy supplies under different climatic conditions. The calculation formulas for specific CO_2_ (%), NO (mg/m^3^), NO_2_ (mg/m^3^) and SO_2_ (mg/m^3^) emissions are as follows:

A. Mass concentration expression: the mass of pollutants contained in each cubic meter of air, i.e. mg/m^3^;

B. Volume concentration expression: the volume of pollutants contained in one million volumes of air, that is ppm, 1% = 10^4^ ppm.

(1) The conversion formula between gas volume concentration and mass concentration (% converted to mg/m3) is shown in (1), referring to the indoor air quality standard GB/T 18,883–200^32^.

1 $$\rho = \frac{{\text{M}}}{22.4}\phi \left[ {\frac{{T_{0} }}{{T_{0} + T}}} \right]\frac{P}{Ba}$$

In the formula, *p*—gas mass concentration, unit: mg/m3; M—gas molecular weight, unit: g/mol, 22.4—gas molar volume under standard conditions, unit: L/mol; $\varphi$—gas volume concentration under standard conditions, unit: L/m3 (ppm), T_0_—thermodynamic temperature is 273.15, unit: K, *T*—gas temperature, unit: K; *P*—actual pressure, unit: Pa; Ba—standard atmospheric pressure is 101325 Pa.

(2) C The hourly flue gas flow rate is calculated as shown in formula (2), referring to White, F. M.’ s Fluid Mechanics book^33^.

2 $$V_{0} = \pi r^{2} vh$$

where, *V*_*0*_ represents the flue gas flow rate, π represents pi, r represents the radius of the chimney, *v* represents the flue gas flow rate, and *h* represents time;

(3) The flue gas flow rate is converted to the standard state flow rate as shown in Eq. (3) with reference to White, F. M.’s Fluid Mechanics book^33^.

3 $$V_{1} = \frac{{P_{{}} V_{0} T_{{0}} }}{{\left( {T_{{1}} + T_{{0}} } \right){\text{Ba}}}}$$

where, *V*_*0*_ represents the flue gas flow rate, unit: m^3^/h; *V*_*1*_ represents the flue gas flow rate under standard conditions, unit: m^3^/h; *P* represents the pressure under actual conditions, unit: Pa; *T* represents the gas temperature, unit: K; Ba represents the pressure under standard conditions is 101,325 Pa, T_0_ represents the temperature under standard conditions is 273.15;

(4) The total emission of the measured pollutant concentration at a given time is calculated as shown in Eq. (4), referring to the Fluid Mechanics book by White, F. M.^33^.

4 $$m_{A} = V_{1} \rho t{10}^{{ - 6}}$$

where: *m*_A_ is the total emissions of major gaseous emissions, unit: kg; *V*_*1*_ is the flue gas flow rate, unit: m^3^/h; *ρ* is the gas mass concentration, unit: mg/m^3^; *t* is the emission time, unit: s.

The example of the above calculation formula is based on the calculation of the total 5-h CO_2_ emissions of the first phase of a biomass-fueled roasting room, as shown below:

The first stage was selected for 5 h, the average emission concentration of CO_2_ was 0.88%, the smoke temperature was 35.6 °C, the air pressure was 80.1 kPa, the molar volume at standard temperature and pressure under the standard condition was 22.4 L/mol, the molar mass of CO was 44 g/mol, 1% = 10,000 ppm, the flow rate was 57.6 m^3^/h, and the standard flow rate was 40.3 m^3^/h. The smoke pipe has a diameter of 16 cm, a radius of 8 cm, a cross-sectional area of 0.02 m^2^ and a flow rate of 0.8 m/s.

The CO_2_ gas volume concentration was converted to a mass concentration (% converted to mg/m^3^) using Eq. (1).

$$\rho = \frac{{\text{M}}}{22.4}\phi \left[ {\frac{{T_{0} }}{{T_{0} + T}}} \right]\frac{P}{Ba}{ = }\frac{{{44}}}{22.40}{*10000*0}{\text{.88}}\left[ {\frac{{{273}{\text{.15}}}}{{{273}{\text{.15}} + {35}{\text{.60}}}}} \right]\frac{{{80}{\text{.1}}}}{{{101}{\text{.325}}}}{ = } {12089}{\text{.20 mg/m}}^{{3}}$$

Calculate the hourly flue gas flow rate by using Eq. (2).

$$V_{0} = \pi r^{2} vh{ = 3}{\text{.14*0}}{.08}^{{2}} *0.8*3600 = 57.87{\text{m}}^{{3}} {\text{/h}}$$

The CO_2_ flue gas flow rate is converted to the flue gas flow rate in the standard condition by Eq. (3).

$$V_{1} = \frac{{P_{{}} V_{0} T_{{0}} }}{{\left( {T_{{1}} + T_{{0}} } \right){\text{Ba}}}}{ = } \frac{{{80}{\text{.10*57}}{.87*273}{\text{.15}}}}{{\left( {{35}{\text{.60}} + {273}{\text{.15}}} \right){*101}{\text{.325}}}}{ = 40}{\text{.47 m}}^{{3}} {\text{/h}}$$

The total amount of CO_2_ emission in the first stage is calculated by Eq. (4).

$$m_{A} = V_{1} \rho t{10}^{{ - 6}} { = 12089}{\text{.20*40}}{.47*5/1000000 = 2}{\text{.45 kg}}$$

## Results and discussion

### Comparison of main gas emissions at each stage in different energy curing rooms

#### Analysis of main gas emission concentrations at each stage in curing rooms using different fuels

##### Analysis of main gas emission concentrations at each stage of biomass fuel curing room under standard working conditions

**Fig. 5:**
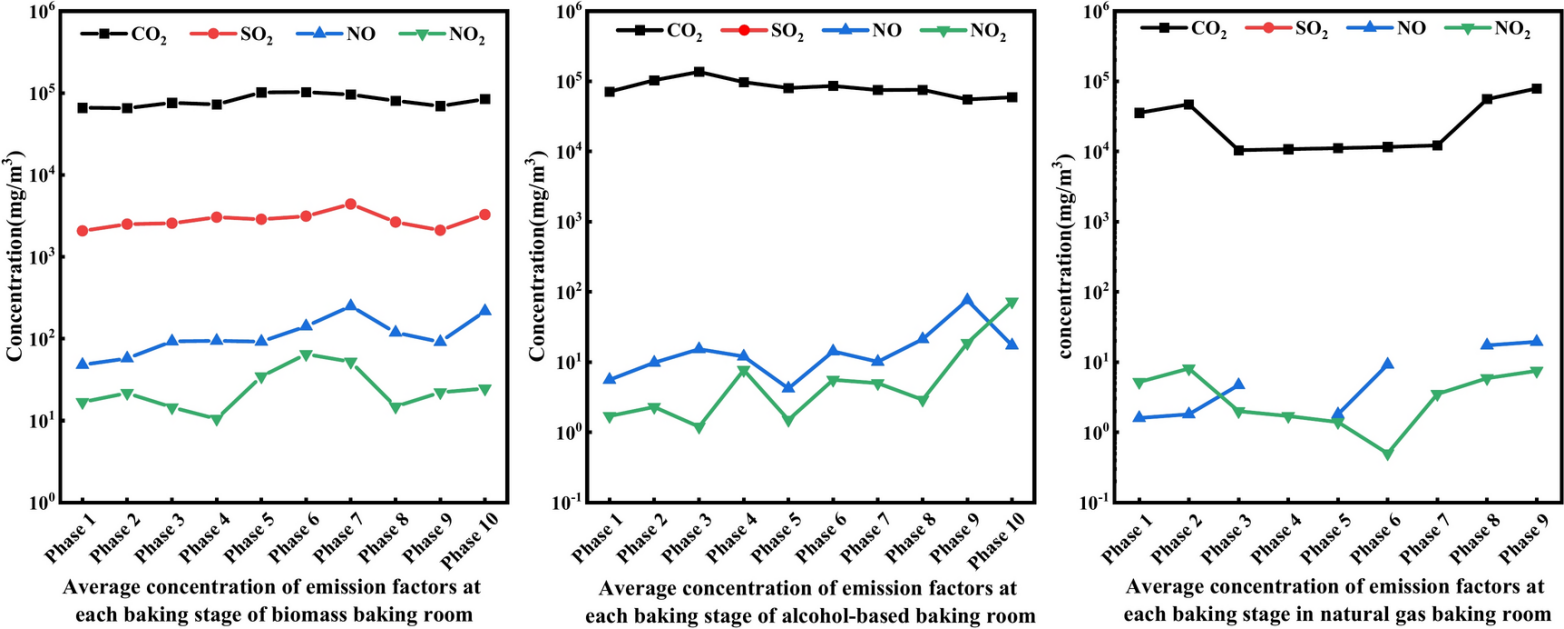
Average concentration of main gas emissions at each stage of curing rooms with different fuels.

It can be seen from Fig. 5, during the entire combustion stage, CO_2_ emission concentration was the highest, followed by NO and SO_2_, and NO_2_ was the lowest. The average emission concentration of CO_2_ was relatively stable before the fourth stage, and began to rise significantly from the fifth stage. This was because in the middle stage of combustion, the decomposition and oxidation of the fuel intensified, resulting in a significant increase in CO_2_ emissions. The average concentrations of SO_2_ and NO showed a slow increasing trend throughout the combustion process, which may be related to the gradual release and combustion of S and N elements in the biomass fuel. The average concentration of NO_2_ was the lowest among all pollutants, but its fluctuation range was the largest, indicating that the generation and release of NO_2_ during biomass combustion was closely related to the properties of the fuel, combustion temperature and redox conditions.

##### Analysis of main gas emission concentrations at each stage of alcohol-based fuel curing room under standard working conditions

It can be seen from the middle of Fig. 5, during the entire combustion stage, CO_2_ emission concentration was the highest, NO and NO_2_ were the lowest, and there is no SO_2_ emission. The average concentration of CO_2_ was relatively stable during the entire combustion process, indicating that during the combustion of the alcohol base, the oxidation reaction of the carbon element was relatively stable and the amount of CO_2_ generated will not change significantly. NO and NO_2_ fluctuated during the combustion process, with the average concentration of NO reaching a minimum of 4.2 mg/m^3^ in the fifth stage; The average concentration of NO_2_ fluctuated repeatedly in the first five stages because the combustion conditions changed greatly during this stage, resulting in the suppression of nitrogen oxides. The emission concentrations of NO and NO_2_ gradually increased from the sixth stage to the tenth stage, indicating that with increasing combustion of the fuel, the oxidation reaction of the N element gradually strengthened, resulting in an increase in the generation of N and O compounds.

##### Analysis on emission concentration of main gas emissions at each stage of natural gas fuel baking room under standard working conditions

It can be seen from the right of Fig. 5, CO_2_ emission concentration changes relatively steadily during the entire operation of the curing room, indicating that the natural gas was burned fully. The emission concentration variation trend of NO_2_ was quite similar to that of CO_2_. Unlike CO_2_, the concentration of NO_2_ reached its minimum value of 0.5 mg/m^3^ in the sixth stage, which may be due to changes in combustion temperature, oxygen supply and fuel decomposition rate. The NO emission concentration changes relatively steadily during the entire operation of the oven. The NO emission in the fourth and seventh stages was 0 mg/m^3^, indicating that conditions for the formation of N and O compounds did not met in this stage. The NO concentration in the remaining stages was maintained between [1.6–19.4] mg/m^3^. During the operation of the natural gas fuel baking room, the generation of NO and NO_2_ was relatively small. This is because the proportion of O element in the composition of natural gas is 0, resulting in the redox reaction of internal N and O compounds, which requires external O element to proceed.

#### Analysis of main gas emissions at each stage of curing rooms using different fuels

##### Analysis of main gas emissions at each stage of biomass fuel curing room

**Fig. 6:**
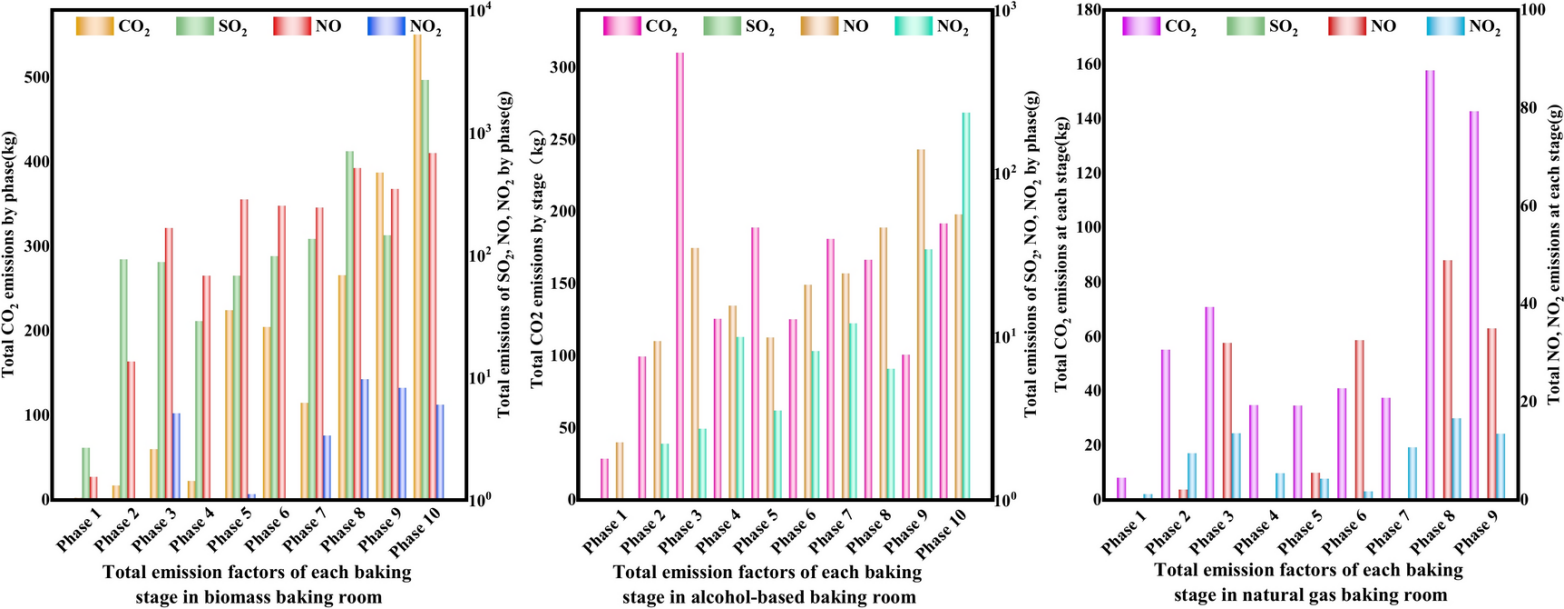
Emissions of main gas emissions at each stage of baking rooms with different fuels.

It can be seen from the left of Fig. 6 thatCO_2_ emissions experienced obvious fluctuations throughout the process, reaching a peak in the fifth stage, then gradually decreasing, and gradually increasing again after the seventh stage until it reached a value of 541 kg in the tenth stage. The total emission of SO_2_ and NO showed relatively gentle fluctuations throughout the combustion process. The emission of SO_2_ and NO gradually reached their peak of 2.678 kg and 0.675 kg in the tenth stage. This is because the release of S and N elements is a relatively slow and continuous process. NO_2_ emissions are almost negligible in the first six combustion stages, reaching a maximum value of 0.0096 kg in the eighth stage and then gradually decreasing. This is because the generation of NO_2_ is usually closely related to the combustion conditions and the balance of redox reactions.

##### Analysis on main gaseous emissions at each stage of alcohol-based fuel curing room

It can be seen from the middle of Fig. 6, during the entire combustion stage, CO_2_ emission concentration was the highest. NO and NO_2_ were the lowest, and there was no SO_2_ emission. CO_2_ emission was relatively stable throughout the combustion process, indicating that during the combustion of alcohol-based products, the amount of CO_2_ generated will not change significantly due to changes in combustion time or stage. NO and NO_2_ emissions fluctuate during the combustion process, but the overall trend was upward. There are slight fluctuations in the first five stages, and as combustion continues, the sixth stage begins to increase gradually, indicating that as the fuel burns further, the oxidation reaction of the N element gradually increases, resulting in an increase in the generation of N and O compounds.

##### Analysis of the emission of main gas emissions at each stage of natural gas fuel baking room

It can be seen from the right of Fig. 6, the emission factors of natural gas fuel baking room gradually increased in the initial stage and reached the first significant peak in the third stage. As the combustion process continues, the total emissions of the emission factor begin to gradually decrease. This trend shows that as the fuel is consumed, the combustion reaction in the drying room gradually stabilizes, and the generation rate of emission factors slows down, resulting in reduced emissions. As the drying room entered the eighth stage of operation, the total emission of emission factors increased significantly again and reached the maximum value of the entire stage, with CO_2_ emissions of 157.78 kg, NO emissions of 0.0489 kg and NO_2_ emissions of 0.0167 kg. At this point, the fuel in the drying room may have been nearly completely burned, resulting in an increase in the generation of emission factors.

#### Comparison of main gas emissions per cubic meter during the baking life cycle of different fuel baking rooms

**Fig. 7:**
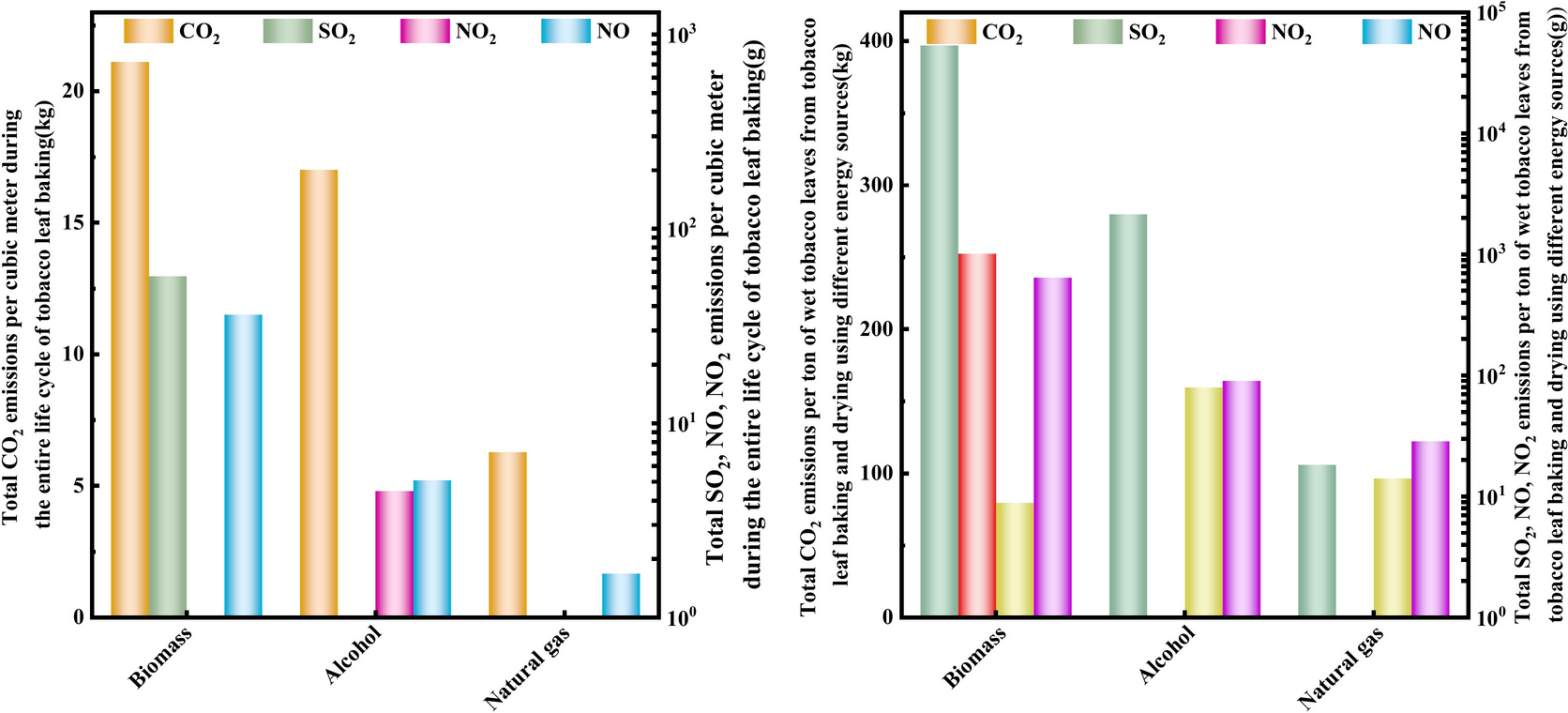
Total emissions per cubic meter of each major gas emission unit during the tobacco leaf curing life cycle in different energy curing rooms (left) Total emissions per ton of wet tobacco leaf (right).

As can be seen from the left of Fig. 7, the total emissions per cubic meter of each emission factor of different energy sources during the tobacco baking life cycle are biomass, alcohol-based, and natural gas from most to least. The highest total emissions of CO_2_, SO_2_, NO and NO_2_ per cubic meter was from biomass, which were 21.1 kg, 0.0567 kg, 0.0361 kg and 0.0005 kg respectively. Specifically, biomass had the most types of emission factors, and the emission of each pollutant is relatively large. This phenomenon is related to the composition of biomass fuel and its combustion process. Total NO_2_ emissions per cubic meter from alcohol and natural gas were almost zero because the proportion of N and O elements in alcohol and natural gas fuels was very small, so related redox reactions may not occur during the combustion process. As for SO_2_, since natural gas and alcohol fuels contain zero S element, almost no redox reaction can occur and no SO_2_ was discharged. Therefore, natural gas and alcohol produce far fewer harmful substances when burned than coal, demonstrating their advantages in reducing air pollution.

According to Fig. 7 on the right, total CO_2_ emissions generated per ton of wet tobacco leaves during the baking process in biomass and alcohol fuel barns were 396.67 kg and 279.71 kg, respectively. However, natural gas showed a significant advantage among all fuels, with its CO_2_ emission of only 105.97 kg per ton of wet tobacco, indicating that the use of natural gas as a fuel has significantly reduced CO_2_ emissions. The maximum SO_2_ emission from biomass was 1.008 kg, while the SO_2_ emission from alcohol and natural gas was 0, because natural gas and alcohol fuels contain 0 S element, so redox reaction can hardly occur. The NO and NO_2_ emissions of biomass were the highest, which were 0.641 kg and 0.079 kg respectively. Natural gas had no NO and the lowest NO_2_ emissions because the proportion of O element contained in natural gas fuel was 0, and the NOX produced is caused by the redox reaction with O element in the air.

### Analysis of main gas emissions during yellowing, color fixing and rib drying periods in curing rooms with different energy sources

#### *Analysis of CO*_*2*_* emissions from different energy curing rooms during the yellowing, color fixing and dry period of tobacco leaves*

**Fig. 8:**
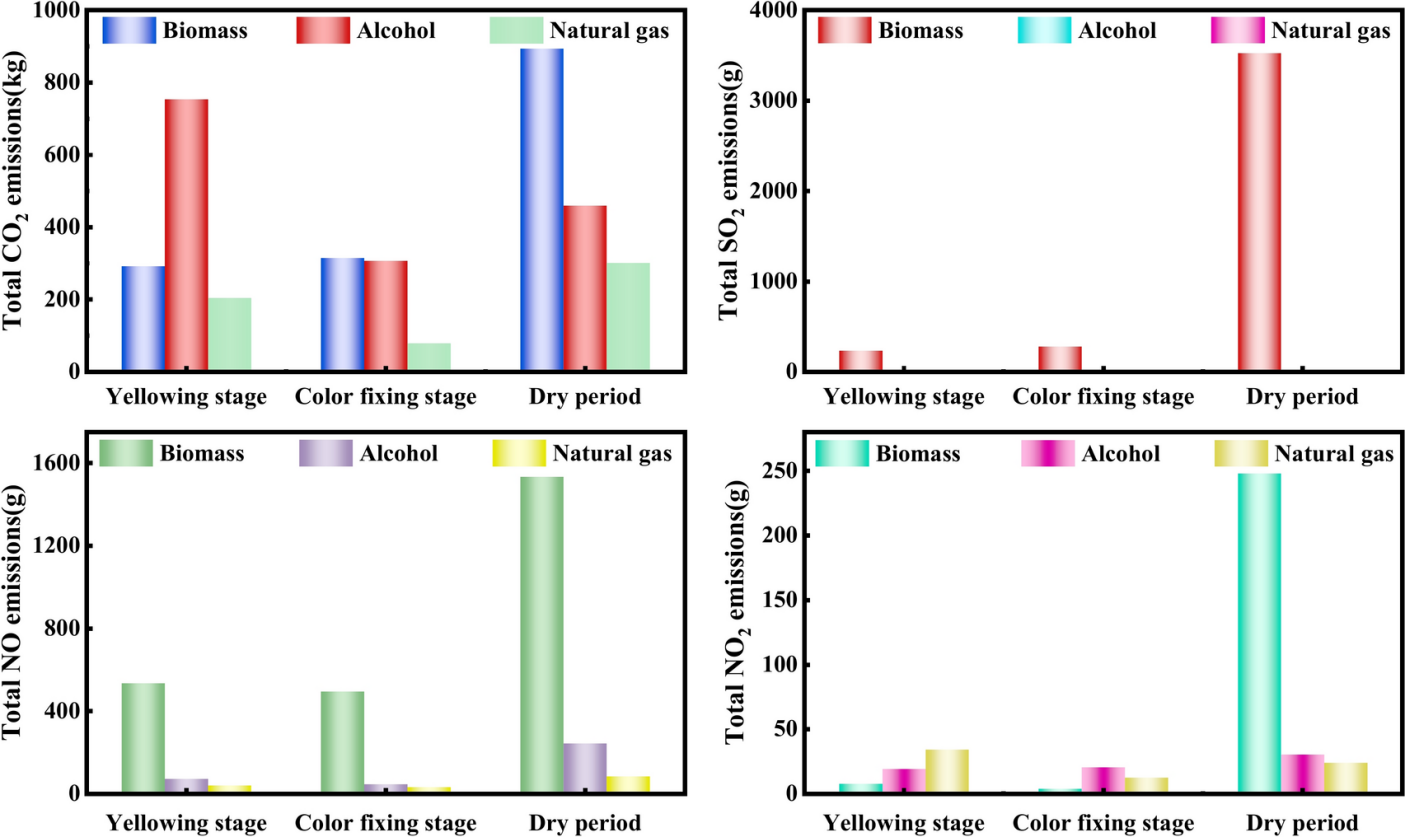
Emissions of CO_2_, SO_2_, NO and NO_2_ in tobacco leaf yellowing, color fixing, and dry period in different energy curing rooms.

As shown in the upper left of Fig. 8, the CO_2_ emission of the bio-roasting room in the tobacco yellowing, color fixation and dry tendon stages was gradually increasing, in which the most CO_2_ emission in the tobacco dry tendon stage was 892.94 kg, which is mainly due to the long roasting time and high energy usage in this stage. Alcohol based roastery had the highest CO_2_ emission of 753.09 kg during the yellowing stage of tobacco. natural gas roastery had the highest CO_2_ emission of 300.53 kg during the dry tendon stage of tobacco.

#### *Analysis of SO*_*2*_* emissions from different energy curing rooms during the yellowing, color fixing and dry period of tobacco leaves*

As shown in the upper right corner of Fig. 8, the SO_2_ emissions from the biomass curing room gradually increased during the tobacco leaf yellowing period, color fixing period and rib drying period, with an increase rate of 16.4% and 92.07% respectively. The maximum SO_2_ emission during the tobacco leaf rib drying period was 35.23 kg, because the baking time in this stage is long and the drying box requires a high temperature. The fuel used in alcohol and natural gas curing rooms does not contain sulfur element, so these two curing rooms do not emit SO_2_.

#### Analysis of NO emissions from different energy curing rooms during the yellowing, color fixing and dry period of tobacco leaves

As shown in the lower left of Fig. 8, the NO emissions from biomass, alcohol and natural gas curing rooms are all the highest during the dry period, at 1.53 kg, 0.242 kg and 0.084 kg, respectively. The NO emissions from alcohol-based and natural gas curing rooms are much less than those from biomass, because alcohol and natural gas contain less N and O elements, and redox reactions cannot fully occur.

#### *Analysis of NO*_*2*_* emissions from different energy curing rooms during the yellowing, color fixing and dry period of tobacco leaves*

As shown in the lower right corner of Fig. 8, the biomass and alcohol curing rooms had the highest NO_2_ emissions during the tobacco leaf dry shank period, at 0.248 kg and 0.0302 kg respectively. This is mainly because the tobacco leaf dry shank requires a higher baking temperature and a large amount of fuel, resulting in the highest NO_2_ emissions in this stage. The maximum NO_2_ emission during the yellowing period of the natural gas baking room was 0.034 kg. The NO content in the yellowing and color fixing stages of each energy source is relatively low because the baking time in these two stages is relatively short. The high emission during the drying period is due to the long baking time and high temperature required in the drying oven during this stage.

### Analysis on the total amount of main gas emissions from ovens with different energy sources

**Fig. 9:**
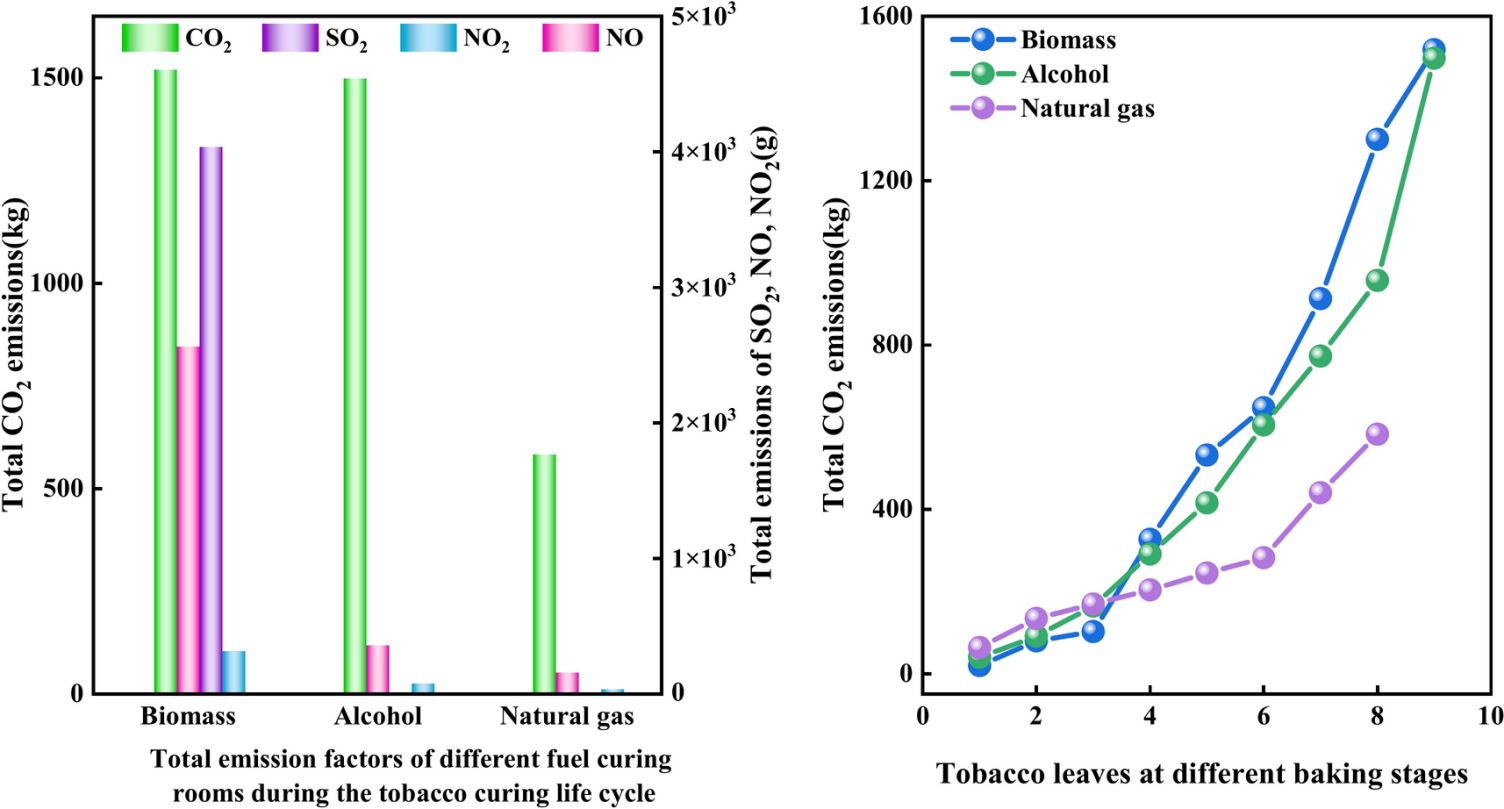
Total emissions of major gaseous emissions from different fuel curing rooms during the tobacco curing life cycle (left) and total CO_2_ emissions at different curing stages (right).

According to the left of Fig. 9, it can be seen that the emissions of life cycle emission factors of baking in different fuel roasters are biomass, alcohol and natural gas, in descending order. **From the analysis of CO**_**2**_** emissions:** Biomass emissions were the highest at 1518.82 kg, followed by alcohol-based and natural gas. This was because biomass contained high levels of C or O elements, which resulted in violent redox reactions during combustion. The other fuels contained lower levels of C or O. **From the analysis of SO**_**2**_** emissions:** The highest biomass emission was 4.035 kg, while the emissions of alcohol and natural gas were 0. This is because the S content of alcohol and natural gas fuels was 0, and redox reactions cannot occur. **From the analysis of NO emissions:** Biomass emissions were the highest at 2.562 kg, followed by alcohol-based and natural gas emissions at 0.156 kg. This is because biomass fuels contain high levels of N and O elements, while natural gas fuels contain 0 O. Although the alcohol fuel contains a high level of O, it only contains 0.06% N, which is why this phenomenon occurs. **From the analysis of NO**_**2**_** emissions:** Biomass had the highest emission, natural gas had the lowest emission of 0.035 kg, and the reason for NO_2_ was the same as NO. The results show that natural gas had the lowest emission among various emission factors.

According to the right side of Fig. 9, it can be seen that the CO_2_ emissions of various pollutants increase with the increase of baking time. The emission slope of the emission factor shows that the CO_2_ emissions of the biomass baking room are relatively stable in the early stage of baking, accelerate in the middle stage, and are the fastest in the late stage, with the highest value of 1518.82 kg. The CO_2_ emission rate of alcohol and natural gas baking rooms is relatively slow, and the emission reaches the maximum in the tenth stage. It may be that they use mechanical feeding method, and the heating is easy to control, while traditional manual coal adding is difficult to accurately control the fuel demand and the heat loss is serious. It can be seen from this that natural gas curing rooms had the lowest CO_2_ emissions and can subsequently become the main energy source for tobacco leaf curing.

### Energy consumption economic evaluation

According to the General Rules for Calculation of Comprehensive Energy Consumption (GB/T2589-2008), fuel and electricity consumption are converted into standard coal consumption. The standard coal conversion coefficient of biomass is 0.5250, the standard coal conversion coefficient of alcohol base is 1.09, and the standard coal conversion coefficient of natural gas is 1.2143. It can be seen from Table 3 that the ratio of fresh smoke to dry smoke after baking is approximately 7:1. In the case of the same proportion of fresh tobacco leaves, it was found that the natural gas consumption was the lowest and the biomass consumption was the highest at 682.50 kg. The power consumption of baking a furnace of tobacco with different fuels was about 250 kw·h. This leads to the conclusion that natural gas should be preferred as the preferred fuel for roasting as natural gas roasters are cheaper, more energy efficient and emit the least amount of pollutants.

**Table 3:** Weights of dry and fresh tobacco and electricity consumption for the full life cycle of roasting in roasting rooms with different fuels.

Energy	Fresh cigarette weight /kg	Dry cigarette weight /kg	Fuel consumption per furnace /kg	Power consumption / (kW·h)	Equivalent to standard coal /kg
Biomass	4190.40	588.39	1300.00	249.00	682.50
Alcohol	4000.00	561.65	467.00	255.00	509.03
Natural gas	5500.00	772.27	477.13	260.00	579.38

**Fig. 10:**
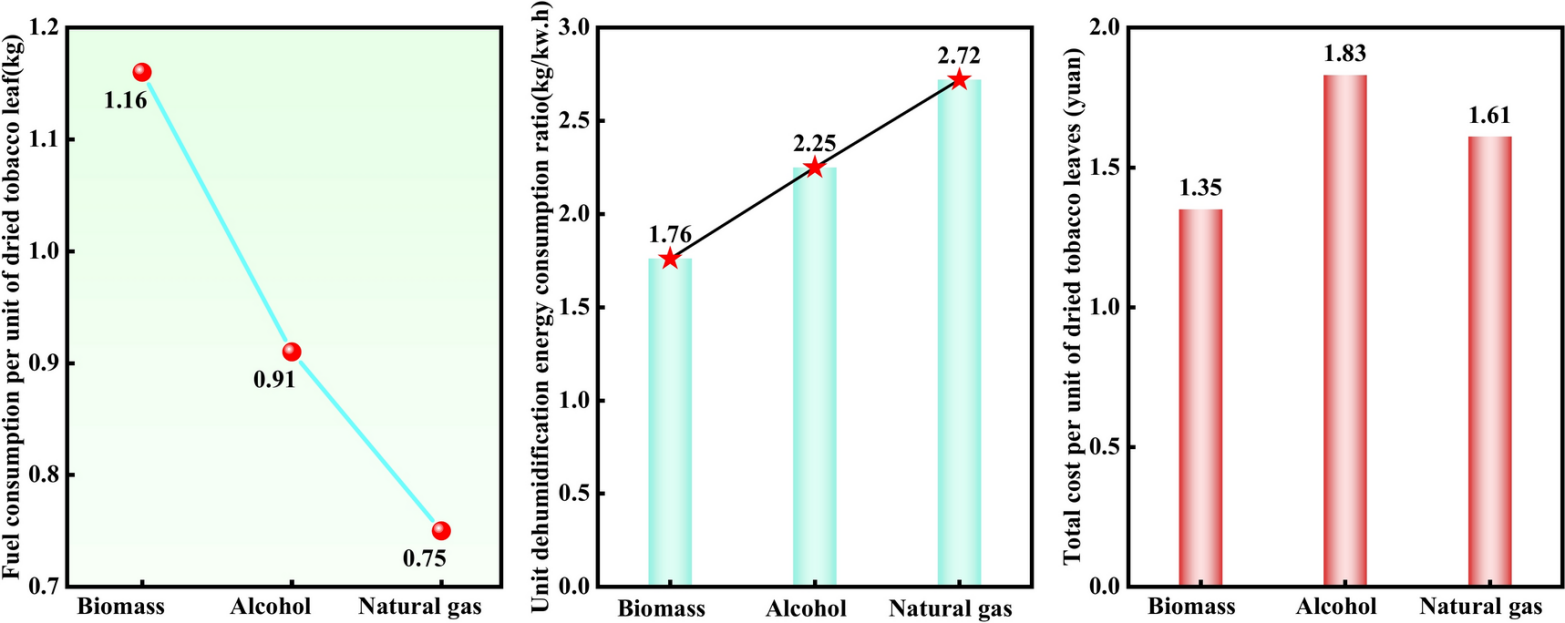
Unit dry tobacco fuel consumption of different fuel curing rooms (left) Unit dehumidification energy consumption ratio (middle) Unit dry tobacco leaf cost over the entire life cycle (right).

As can be seen from the left of Fig. 10, the unit dry tobacco fuel consumption of the natural gas curing room was 0.75 kg, which was 17.58% less than that of the alcohol curing room and 35.34% less than that of the biomass pellets. From the perspective of unit dry tobacco fuel consumption, the natural gas curing room has obvious energy-saving advantages.

It can be seen from Table 4 and Fig. 10 that the moisture content of fresh tobacco in natural gas curing barns is significantly higher than that in curing barns using other fuels. When the weight of fresh tobacco leaves is the same, the power consumption of natural gas curing barns is lower. The unit dehumidification energy consumption ratios of different fuels were 1.76, 2.25 and 2.72, respectively. It is found that the unit energy consumption dehumidification capacity of the natural gas baking room is much higher than that of biomass and alcohol, and its value was 1.55 times that of biomass and 1.2 times that of alcohol. It can be seen that the natural gas curing room has significantly improved the efficiency of tobacco leaf curing, and the drying and energy-saving advantages are obvious.

**Table 4:** Unit dehumidification energy consumption ratio of different fuel baking rooms.

Energy	Water loss /kg	Converted power consumption / (kW·h)	Unit dehumidification energy consumption ratio
Biomass	3602.01	2047.50	1.76
Alcohol	3438.35	1527.09	2.25
Natural gas	4727.73	1738.13	2.72

It can be seen from Table 5 and Fig. 10 on the right that the unit dry tobacco fuel costs of biomass curing room, alcohol-based curing room and natural gas curing room were 1.16 yuan, 1.63 yuan and 1.46 yuan respectively, and the unit dry tobacco electricity costs were 0.19 yuan, 0.20 yuan and 0.15 yuan respectively. Among them, the biomass curing room has the lowest unit dry tobacco fuel cost, and the natural gas has the lowest unit dry tobacco electricity cost. The highest unit dry tobacco leaf cost during the life cycle of baking in flue-curing rooms using different fuels was 1.83 yuan for alcohol tobacco, the lowest was 1.35 yuan for biomass, and 1.61 yuan for natural gas. Although biomass curing room had the lowest cost for drying tobacco leaves, its combustion produces more SO_2_, NO and NO_2_. Therefore, in order to develop tobacco baking in a sustainable, environmentally friendly, energy-saving and efficient direction, natural gas and alcohol fuels are preferred because they have obvious advantages in reducing costs and increasing efficiency.

**Table 5:** Cost per unit of dried tobacco leaves in flue-curing barns with different fuels.

Energy	Fuel consumption /kg	Fuel cost/yuan	Total power consumption / (kW·h)	power consumption / (kW·h)	Electricity bill for dry cigarettes /(yuan/kg)	Dried tobacco leaf cost /(yuan/kg)
Biomass	1.16	1.16	249	0.42	0.19	1.35
Biomass	0.91	1.63	249	0.44	0.20	1.83
Natural gas	0.75	1.46	249	0.32	0.15	1.61

## Conclusion and discussion

### Comparative analysis and discussion with previous studies

In this section, comparisons were made between the current study and relevant research results in recent years considering the economic evaluation of the main gas emissions and energy consumption of the clean energy tobacco leaf baking life cycle. Table 6 can more intuitively demonstrate the advantages of the current study, in which the optimal value is marked in bold. Specific details are summarized as follows.

**Table 6:** Statistical comparison of this paper with the literature on different fuel curing houses published in recent years in terms of main gas emissions, energy consumption, economy, yellowing period, color fixation period and steel bar drying period.

Author	Fuel	Emission factors	Energy consumption	Economical	Yellowing, color fixation and dry period	Main conclusions
Liu ^12^	Alcohol, biomass, coal	CO/SO_2_/ NO_x_	Fuel usage, power consumption	Electricity, labor and fuel cost	No	The NO_x_ and CO emissions of alcohol-based fuel baking were reduced by 84.7% and 99.7%, respectively
Lan ^24^	Biomass, coal	CO_2_/SO_2_/ NO_x_	Fuel quantity	Tobacco leaf price	No	The exhaust gas emissions from biomass drying rooms are much lower than those from conventional drying rooms
Guo ^28^	Alcohol, biomass, coal	Yes	Power and fuel consumption	Electricity costs, fuel costs	Statistics of baking time for each period	All three alternative energy sources are superior to lignite in terms of pollutant emissions
Zhang ^30^	Coal	No	No	No	Statistics of baking time for each period	The yellowing period is long, while the color fixing period and the drying period are short
Kaifeng Shuai ^34^	Biomass, coal	CO_2_/SO_2_/ CO	fuel consumption	No	No	The exhaust gas concentrations from coal-fired and biomass drying rooms have dropped significantly
**Proposed study**	**Biomass, alcohol, natural gas**	**CO**_**2**_**/SO**_**2**_**/NO/NO**_**2**_	**Fuel consumption per furnace, power consumption, unit dehumidification energy consumption, fuel cost**	**Electricity cost, dried tobacco leaf cost, fuel consumption cost**	**Statistical analysis of baking time and main gas emissions in each period**	**Natural gas and alcohol products can play a clear role in energy conservation, emission reduction, cost reduction and efficiency improvement**

In Table 6, a comparative analysis is made with among multiple studies on curing houses using different fuels published in recent years in terms of major gas emissions, energy consumption, economy, yellowing period, color fixing period, and tendon drying period. In general, this paper comprehensively analyzes the main gas emissions and emissions at various stages, throughout the entire process, including the yellowing period, the color fixing period, and the drying period of tobacco leaves by curing tobacco leaves with biomass, alcohol-based, and natural gas fuels, and conducts a comprehensive evaluation of the energy consumption and economy. In terms of major gas emissions: Zhaoyu Liu^12^ only tested the main pollutant content of CO, SO_2_ and NO_x_ in flue gas. Shubing Lan^24^ found that burning 1 ton of coal would release 2620 kg of CO_2_, 8.5 kg of SO_2_ and 7 kg of NO_X_. In this study, biomass, the fuel with the most gas emissions, consumed 1.3 tons of fuel during the whole life cycle of baking, but only produced 1518.82 kg of CO_2_, 4.035 kg of SO_2_ and 2.562 kg of NO_X_. Kaifeng Shuai^34^ only conducted statistics on the CO and CO_2_ emission concentrations before and after the coal-fired desulfurization and dust removal renovation of the baking room. Dayang Guo^28^ only counted the emission concentrations of the main pollutants in flue gas, such as CO_x_, SO_2_ and NO_x_, during the baking process of different energy sources. The current study selected three clean energy sources, namely biomass, alcohol-based, and natural gas, and fully studied the emission concentrations and emissions of the main gases (CO_2_, SO_2_, NO and NO_2_) at each stage under the baking of each clean energy source, as well as the emissions during the entire baking process. From the perspective of energy economy: This study provides an overall analysis of the fuel consumption per furnace, power consumption, unit dehumidification energy consumption, fuel cost, electricity cost, dry tobacco leaf cost and fuel consumption cost, which is richer and more comprehensive than the analyses in the above-mentioned references, i.e.,^12,24,28^ and^30^. In terms of the yellowing stage, color fixing stage and dry-stem stage: Dayang Guo^28^ and Fusheng Zhang^30^ only counted the baking time of alcohol-based, biomass and coal. This study not only counted the baking time of each stage, but also statistically analyzed the main gas emissions of each stage. Results showed that CO_2_, SO_2_, NO and NO_2_ emissions gradually increased during the yellowing stage, color fixing stage and dry-stem stage of tobacco leaves, and the highest emissions were in the dry-stem stage of biomass curing barn. There was no SO_2_ emission in alcohol-based and natural gas curing barns, indicating that the curing process can be optimized by optimizing the combustion process and adjusting the curing temperature and humidity in specific stages. Compared with the literature in Table 6, this study shows that natural gas and alcohol genes, with their lower gas emissions and good economy, can play a significant role in energy conservation, emission reduction, cost reduction and efficiency improvement.

Through research, it was found that the types and contents of elements contained in coal fuel are relatively more than the above three energy sources. Therefore, more pollutants such as CO_X_, NO_X_, SO_2_, etc. will be produced during the combustion process. If the coefficient of converting raw coal into standard coal is 0.7143, when baking the same proportion of fresh tobacco leaves, compared with the above three energy sources, the coal consumption is higher, the electricity consumption is higher and the unit dehumidification energy consumption ratio is lower. Electric curing rooms are suitable for small-scale, high-precision and high-environmental requirements, but in large-scale and high-temperature curing rooms, there are problems of high operating costs and strong dependence on power supply. Compared with coal-fired and electric curing rooms, biomass, alcohol-based and natural gas curing rooms are more environmentally friendly, safer and more efficient. This study’s comparative evaluation found that natural gas and ethanol have lower initial investments and can be used as reliable alternative energy sources for subsequent tobacco baking, but there are still high fuel costs and certain CO_2_ and NO_x_ emission problems. At present, new energy heat pump drying rooms have obvious advantages in energy saving and environmental protection, but the initial investment is high and the dependence on electricity is strong. If the power supply is unstable or the electricity price is high, it may affect its economy. In addition, the efficiency of the heat pump system is greatly affected by the external environment (such as temperature and humidity). Tobacco baking takes about 3 months a year, and it is impossible to achieve quick profits, but long-term operating costs are low and the economy benefits is better. In summary, coal-fired and electric curing rooms have problems such as severe environmental pollution and high operating costs. New energy heat pump curing rooms are suitable for long-term energy conservation and emission reduction, especially if clean electricity sources can be used. Their benefits in economy and energy conservation seem more prominent. However, natural gas, alcohol-based and biomass curing rooms have great advantages in short-term investment, rapid profitability, energy conservation and environmental protection. However, there are also potential obstacles in terms of economy, regulation and technology. For natural gas, the economic barriers include high pipeline construction costs and high gas storage cost pressure, the regulatory barriers include complex pipeline separation policies and approval processes, and the technical barriers include reliance on pipeline coverage and intelligent upgrade requirements. For alcohol-based fuel, the economic barriers include safety hazard costs. The regulatory barriers include lack of industry standards and policy restrictions, and the technical barriers include high safety risks and misuse risks. Both natural gas and alcohol-based need to deal with environmental compliance costs and technical upgrade pressures. In future work, we will focus on comprehensive baking performance and economy of the heat pump baking room to obtain a better baking solution and achieve a balance between energy saving, environmental protection and rapid profitability.

### Analysis and discussion of relevant policy impacts

In tobacco baking, transition from biomass fuel to clean energy (such as alcohol-based and natural gas) is not only an inevitable path for technological iteration, but also a key link in the policy-driven green transformation of agriculture. The potential policy impact of this transition is analyzed from the following three aspects.

- (1). Environmental policy

Although biomass fuel is lower in carbon emissions than coal (carbon emissions are reduced by about 50%), its combustion still produces nitrogen oxides and particulate matter pollution, while alcohol-based fuels (such as methanol and ethanol) and natural gas can almost achieve near-zero emissions. This finding bears huge practical implications. For example, in the context of the study, clean drying houses have been subsidized since 2018. By 2024, more than 100,000 houses have been built, reducing sulfur dioxide emissions by more than 1,000 tons. The "14th Five-Year Plan for Renewable Energy Development" clearly requires the acceleration of the development of biogas and the promotion of its application in industry, transportation and other fields. In addition, the natural gas industry chain can coordinate with the treatment of agricultural waste (such as straw and livestock and poultry manure) to reduce open-air burning pollution. At the same time, its carbon neutrality and carbon reduction benefits can be included in the carbon trading system to create additional income for tobacco farmers.

- (2). Economic policy

The cost of biomass fuel is about 50%-60% that of coal, while the long-term operating costs of alcohol-based fuel and biogas are lower. The central government invests 2 billion yuan each year to support biogas projects, while promoting carbon trading mechanisms to convert the carbon reduction benefits of clean fuels into economic benefits. In addition, natural gas projects can drive rural employment. For example, the straw processing plant in Jinning, Yunnan, processes 8 tons of waste per day, forming an industrial chain of recycling and transportation, which helps rural revitalization.

- (3). Energy policy

The localized production of biogas and alcohol-based fuels can reduce dependence on external energy. The 2024 "Guiding Opinions on Renewable Energy Substitution Action" advocated the development of biogas in accordance with local conditions, promoting coordinated treatment of agricultural and forestry wastes, and optimizing the energy consumption structure. In addition, high energy density of alcohol-based fuels and the peak-shaving capacity of natural gas can make up for the intermittent nature of wind and solar energy, which in turn enhances the stability of the energy system. The transformation of biomass to alcohol-based and natural gas requires relevant policies to work together in technology research and development, subsidy mechanisms and carbon market linkage to build a win–win green governance framework for all parties and accelerate clean energy substitution.

## Conclusion

In this study, biomass, alcohol-based and natural gas were used to evaluate the concentration and emission of main gas emissions (CO_2_, SO_2_, NO_2_ and NO) at each stage of the tobacco leaf curing life cycle. Specifically, it analyzes the emission factors (per cubic meter) of these gases, and quantifies emissions during the yellowing stage, color-fixing stage and stem-drying stage in clean energy curing barns as well as comparing the total emissions and energy consumption economics across the entire curing process for different clean energy curing systems. Main findings can be summarized as follows.

1. Under standard working conditions, the CO_2_, NO and SO_2_ emission concentrations of biomass curing rooms at each stage were the highest, and there was no SO_2_ emission in alcohol-based and natural gas curing rooms. The biomass curing room had the highest CO_2_ and NO emissions at each stage and the alcohol-based curing room had the highest NO_2_ emissions. The characteristics and differences of different fuels in terms of emission concentration and emission at each stage were obtained.

2. Compared with alcohol-based fuel and natural gas fuel, biomass fuel has the highest total emissions of CO_2_, SO_2_, NO and NO_2_ per cubic meter, which were 21.1 kg, 0.057 kg, 0.0361 kg and 0.0005 kg respectively.

3. For each ton of wet tobacco leaves baked, biomass fuel had the highest gas emissions, including 396.67 kg of CO_2_, 1.008 kg of SO_2,_ 0.641 kg of NO and 0.078 kg of NO_2_. Natural gas had an obvious advantage among all fuels, with CO_2_ emissions of only 26.7% of biomass and no NO and NO_2_ emissions. Both alcohol-based and natural gas have no SO_2_ emissions, indicating that the use of natural gas demonstrated a significant effect on reducing CO2 emissions.

4. During the yellowing, color fixing and drying stages of tobacco leaves, the emissions of CO_2_, SO_2_, NO and NO_2_ gradually increased. The highest emissions were from the biomass curing barn during the drying stage, which were 1203.85 kg, 35.23 kg, 1.53 kg and 0.248 kg respectively. There was no SO_2_ emission from the alcohol-based and natural gas curing barns because the fuel used did not contain the S element. This shows that the curing process can be optimized by optimizing the combustion process and adjusting the curing temperature and humidity in stages.

5. Comparison of main gas emissions of the whole life cycle of tobacco baking by various clean energy sources shows that biomass had the largest total emissions. From the total CO_2_ emissions of various energy curing rooms, it can be seen that as the curing time increases, pollutant emissions also increase. The highest CO_2_ emissions from biomass were 1518.82 kg, while the CO_2_ emission rates of alcohol-based and natural gas curing rooms were relatively slow, and the CO_2_ emissions from natural gas curing rooms were the lowest at 582.82 kg.

6. The ratio of fresh tobacco to dry tobacco after baking is about 7:1. Under the same ratio, fresh tobacco consumes the least natural gas, consumes less electricity, and has a higher average dehumidification capacity per unit energy consumption.

7. The cost per unit of dried tobacco leaves in biomass curing rooms was 1.35 yuan, which was 22.3% lower than alcohol-based and 16.2% lower than natural gas. Although the cost per unit of dried tobacco leaves in biomass curing rooms was the lowest, its combustion produces more gas emissions.

8. The evaluation of main gas emissions and energy consumption economy at each stage, each change period and the entire tobacco baking stage found that natural gas and alcohol-based gases had low emissions and good economy. They can play a significant advantage in energy conservation, emission reduction, cost reduction and Improve efficiency.

## Data Availability

If anyone wants to obtain data from this study, they should contact Jiang Yonglei (corresponding author), jiangyatas@163.com (E-mail address).
